# Prediction of Rupture by Complete Blood Count in Tubal Ectopic Pregnancies Treated with a Single-Dose Methotrexate Protocol

**DOI:** 10.1055/s-0043-1772485

**Published:** 2023-10-16

**Authors:** Yıldız Akdaş Reis, Arife Akay, Elif Gülşah Diktaş, Merve Özkan, Neslihan Öztürk, Doğukan Özkan, Betül Tokgöz Çakır, Salim Erkaya

**Affiliations:** 1 Obstetrics-Gynecology Department, Etlik Zübeyde Hanım Maternity and Women's Health Teaching and Research Hospital, Ankara, Turkey

**Keywords:** ectopic pregnancy, methotrexate, neutrophil-to-lymphocyte ratio, platelet distribution width, gravidez ectópica, metotrexato, proporção de neutrófilos para linfócitos, largura de distribuição de plaquetas

## Abstract

**Objective** The availability of reliable and inexpensive markers that can be used to determine the risk of rupture during methotrexate (MTX) treatment in ectopic pregnancies (EPs) is considerable. The aim of the present study is to investigate the role of systemic inflammatory markers such as leukocytes (or white blood cells, WBCs), the neutrophil-to-lymphocyte ratio (NLR), and platelet distribution width (PDW), which are among the parameters of the complete blood count (CBC), in the prediction of rupture of EPs under MTX treatment.

**Materials and Methods** A total of 161 patients with tubal EP who underwent a single-dose methotrexate (MTX) protocol were retrospectively analyzed, and the control group (n = 83) included patients cured by MTX, while the ruptured group (n = 78) included patients who were operated on for tubal rupture during the MTX treatment. The features of EP, beta-human chorionic gonadotropin (β-hCG) levels, sonographic findings, and CBC-derived markers such as WBC, NLR, and PDW, were investigated by comparing both groups.

**Results** The NLR was found to be higher in the ruptured group, of 2.92 ± 0.86%, and significantly lower in the control group, of 2.09 ± 0.6%. Similarly, the PDW was higher (51 ± 9%) in the ruptured group, and it was significantly lower a (47 ± 13%) in the control group ( *p* < 0.05). Other CBC parameters were similar in both groups ( *p* > 0.05).

**Conclusion** Systemic inflammation markers derived from CBC can be easily applied to predict the risk of tubal rupture in Eps, since the CBC is an inexpensive and easy-to-apply test, which is first requested from each patient during hospitalization.

## Resumo

**Objetivo** A disponibilidade de marcadores confiáveis e baratos que podem ser usados para determinar o risco de ruptura durante o tratamento com metotrexato (MTX) em gestações ectópicas (GEs) é considerável. O objetivo do presente estudo é investigar o papel de marcadores inflamatórios sistêmicos, como leucócitos (ou glóbulos brancos, glóbulos brancos), a relação neutrófilo-linfócito (NLR) e largura de distribuição de plaquetas (PDW), que estão entre os parâmetros do hemograma completo (hemograma), na predição de ruptura de PEs sob tratamento com MTX.

**Materiais e Métodos** Foram analisados retrospectivamente 161 pacientes com EP tubária submetidas a protocolo de dose única de metotrexato (MTX), sendo que o grupo controle (n = 83) incluiu pacientes curadas com MTX, enquanto o grupo roto (n = 78) incluíram pacientes operadas por ruptura tubária durante o tratamento com MTX. As características de EP, beta-gonadotrofina coriônica humana (β-hCG), achados ultrassonográficos e marcadores derivados de CBC, como WBC, NLR e PDW, foram investigados comparando os dois grupos.

**Resultados** A RNL foi maior no grupo roto, de 2,92 ± 0,86%, e significativamente menor no grupo controle, de 2,09 ± 0,6%. Da mesma forma, o PDW foi maior (51 ± 9%) no grupo roto, e foi significativamente menor a (47 ± 13%) no grupo controle (p < 0,05). Outros parâmetros do hemograma foram semelhantes em ambos os grupos (p > 0,05).

**Conclusão** Marcadores inflamatórios sistêmicos derivados do hemograma podem ser facilmente aplicados para predizer o risco de ruptura tubária na Eps, uma vez que o hemograma é um exame de baixo custo e fácil aplicação, solicitado primeiramente a cada paciente durante a internação.

## Introduction

Ectopic pregnancy (EP) is defined as the implantation of the fertilized ovum outside the uterine cavity, frequently in the fallopian tubes, and it occurs in 2% of all pregnancies. 1 Although the rate of deaths associated with EP have decreased, 9% to 13% of all pregnancy-related deaths are associated with EP, particularly in developing countries. 2 Methotrexate (MTX), a folinic acid antagonist, is preferred as a cost-effective, first-line therapy in tubal EPs if the patient is hemodynamically stable. 3 However, the success rate of the MTX treatment in EP ranges from 65% to 95%. 4 5 6 Sporadically, the failure of the treatment results in rupture of the EP, which can lead to hemodynamic instability or even death caused by intraabdominal bleeding; therefore, it is crucial to predict rupture.

Conditions that lead to faultiness of implantation signals due to reasons, such as tubal damage caused by infection or inflammation, alter tubal transport and microenvironment, increase receptivity in the tubal epithelium, and are thought to predispose to EP, that is, inflammation is an important factor in the formation and maintenance of EP. 7 8 Many studies 7 9 10 have also shown that the expression of several chemokines and cytokines increases in the tube in which the EP is located. Furthermore, complete blood counts (CBCs) were accepted as serial and basic parameters indicative of systemic inflammation and stress. 11 Markers of CBC, such as the neutrophil-to-lymphocyte ratio (NLR) 12 and the platelet-to-lymphocyte ratio (PLR) 13 are known as inexpensive and easily calculable inflammatory markers that correlate with the prognosis of systemic inflammatory diseases.

Rupture of the EP may occur during MTX therapy and may have dramatic consequences. Therefore, there is a need for inexpensive, sensitive, and easy-to-apply markers for patients undergoing MTX therapy. The present study aims to investigate whether CBC-derived markers such as leukocytes (white blood cells, WBCs), NLR, PLR, and platelet distribution width (PDW) predict EP rupture.

## Materials and Methods

We performed a retrospective evaluation of the data of the patients hospitalized between January 1st, 2014, and January 12th, 2019, with the diagnosis of tubal EP in the Early Pregnancy Service of our institution, and who underwent the single-dose MTX protocol. In our clinic, the diagnosis and treatment of EP are made according to the 2018 American College of Obstetricians and Gynecologist (ACOG) guidelines. 3 The diagnosis of tubal EP is made if the β-HCG level is above 1,500-3,500 mIU/mL, an intrauterine pregnancy is not observed and a tubal ectopic focus is observed on transvaginal ultrasonography (TVUSG), or if the level of beta-human chorionic gonadotropin (β-HCG) is below 1,500 mIU/mL to 3,500 mIU/mL, an irregular β-HCG increase is observed below the minimum threshold (35% to 55%) when serial β-HCG concentration measurements are made at 48-hour intervals, an intrauterine pregnancy is not detected, and a tubal ectopic focus is detected on TVUSG in the follow-up. In addition, an MTX protocol consisting of a single dose of 50 mg/m ^2^ (body surface area) was administered intramuscularly in stable EPs that fulfilled the ACOG criteria and had no contraindications. Accordingly, we excluded cases with the following characteristics: intrauterine pregnancy, evidence of immunodeficiency, moderate to severe anemia, leukopenia or thrombocytopenia, sensitivity to MTX, active pulmonary disease, active peptic ulcer disease, clinically significant hepatic dysfunction, clinically significant renal dysfunction, breastfeeding, active infection, and chronic inflammatory diseases (as they affect hematological parameters). Tubal EPs in which the villus structure is proven not to be in the intrauterine cavity by endometrial sampling, or pregnancies with a histopathology report of tubal EP after surgery, that were hemodynamically stable and were compatible with follow-up and were not clinically ruptured were included in the study. Furthermore, in our clinic, the patients followed up according to the ACOG recommendations. The treatment is as follows: in the single-dose MTX regimen, measure the hCG level on days 4 and 7; if the decrease is greater than 15%, measure β-hCG levels weekly until they reach a non-pregnant level but decrease below 15%. If the β-hCG levels flatten or rise during follow-up, a second dose of MTX is administered, and the measurement of the β-hCG level is repeated. After the single-dose MTX protocol, patients who were followed up until the β-HCG level was zero and did not experience rupture were considered cured. The patients who underwent emergency surgery after the single-dose MTX protocol were found to have ruptured from the tubal region and were histopathologically confirmed were considered ruptured.

The sample was divided into group 1 (cases), which included patients who experienced rupture during the MTX treatment, and group 2 (controls), which was composed of patients successfully treated with MTX. Group 1 was composed of 78 patients. Among the patients cured with the single-dose MTX protocol, the cases with the same age, body mass index (BMI), gestational age, baseline β-hCG, and EP size as the cases in group 1 were selected as the control group. Therefore, differences in these parameters were prevented from affecting the CBC. In addition, when creating the control group, if there were two different cases with similar values for these parameters (there were about five cases), both were included in the control group to avoid bias. As a result, the control group (group 2) was composed of 83 patients cured with the single-dose MTX protocol. In addition, EPs caused by assisted reproductive methods were excluded from the study. Patients with a pregnancy of unknown location, those with non-tubal EPs, patients with inflammatory, hematological, or connective tissue disease, as well as smokers, and the cases whose age, BMI, gestational age, baseline β-hCG, and EP size did not match one of the controls were not included in the study. Approval from the local ethics committee and the necessary consent from the patients were obtained for the study.

Moreover, venous blood is routinely taken from each patient in an anticoagulant tube containing ethylenediaminetetraacetic acid (EDTA) at the time of hospitalization, and the CBC performed with the BC-6000 (Mindray, Shenzhen, China) device in the blood laboratory shows a total of 23 parameters. The first hemogram parameters at the time of hospitalization, WBCs, hemoglobin (Hb), platelets (PLTs), mean corpuscular volume (MCV), NLR, PLR, reticulocyte distribution width (RDW), PDW, and mean platelet volume (MPV) were determined for this study

Age, BMI, obstetric history (gravidity, parity, and ectopic pregnancy), previous history of abdominal surgery and pelvic inflammatory disease, levels and follow-ups of β-hCG, diameter and features of EP material, results and failures of treatment modalities, and all other features were obtained retrospectively from patient files and the electronic patient registry system. All of these parameters for both study groups were compared.

The statistical analyses were made with the IBM SPSS Statistics for Windows (IBM Corp., Armonk, NY, United States) software, version 21.0, and we adopted a confidence level of 95%. If the kurtosis and skewness values obtained from the measurements are between +3 and -3, they are considered sufficient for the normal distribution. The categorical variables were expressed as numbers and percentages, and the numerical variables, as minimum, maximum, mean, and standard deviation (SD) values. Statistical significance was set as *p* < 0.05. In two independent groups, the parametric variables were analyzed with the *t* -test and non-parametric variables, with the Mann–Whitney test. The relationships involving the categorical variables were analyzed with the Chi-squared test. Hematologic markers (WBC, Hb, PLT, NLR, PLR, MPV, MCV, PDW, RDW) were expressed as mean and SD values, and the receiver operating characteristic (ROC) curve was used to show the sensitivity and specificity of each marker. The detection value of each biomarker increases until it is close to 1 when the area under the curve (AUC) is greater than 0.5. The Pearson correlation test was used to assess the relationship regarding free peritoneal fluid, NLR, and PDW.

## Results

There were 83 cases in the control group cured with a single-dose MTX protocol and 78 cases in the ruptured group. There was no significant difference in terms of demographic parameters between the groups. History of pelvic inflammatory disease (PID) was significantly higher in the control group (6% versus 0% in the ruptured group; *p* = 0.028) and the odds ratio (OR) was determined as 5. However, the history of abdominal surgery was insignificantly () higher in the ruptured group (39% versus 28% in the control group; *p* = 0.148), and the OR was found to be of 1.6. The demographic characteristics of the groups are presented in Table 1 .

**Table 1:** Demographics of the study groups

		Groups		Chi-squared	*p*	OR
		Control	Ruptured			
		N = 83: n(%)	N = 78: n(%)			
Gravidity	1	26(31.3)	17(21.8)	9.045	0.060	
	2	22(26.5)	14(17.9)			
	3	22(26.5)	20(25.6)			
	4	6(7.2)	16(20.5)			
	> 5	7(8.4)	11(14.1)			
Parity	0	36(43.4)	25(32.1)	4.079	0.253	
	1	28(33.7)	25(32.1)			
	2	15(18.1)	24(30.8)			
	3–4	4(4.8)	4(5.1)			
Number of previous D&C	0	80(96.4)	73(93.6)	0.666	0.415	1.826
	1–3	3(3.6)	5(6.4)			
Number of previous EP	0	73(88)	66(84.6)	0.379	0.538	1.327
	1	10(12)	12(15.4)			
Number of previous stillbirths	0	80(96.4)	76(97.4)	0.147	0.701	0.702
	1–2	3(3.6)	2(2.6)			
Level of schooling	Illiterate	1(1.2)	2(2.6)	2.949	0.566	
	Primary school	18(21.7)	14(17.9)			
	Secondary school	11(13.3)	17(21.8)			
	High school	29(34.9)	22(28.2)			
	University	24(28.9)	23(29.5)			
History of pelvic inflammatory disease	Not available	78(94)	78(100)	4.849	0.028*	0.500
	Available	5(6)	0(0)			
History of abdominal surgery	Not available	59(71.1)	47(60.3)	2.096	0.148	1.621
	Available	24(28.9)	31(39.7)			
Contraceptive method	Not available	72(86.7)	74(94.9)	7.379	0.194	
	IUD	5(6)	3(3.8)			
	Male condom	0(0)	1(1.3)			
	Coitus interruptus	4(4.8)	0(0)			
	COCs	1(1.2)	0(0)			
	BTL	1(1.2)	0(0)			
Application complaint	Not available	14(16.9)	6(7.7)	7.417	0.115	
	Pelvic pain	13(15.7)	14(17.9)			
	Vaginal bleeding	39(47)	32(41)			
	Pelvic pain + vaginal bleeding	9(10.8)	19(24.4)			
	Menstrual delay	8(9.6)	7(9)			

Abbreviations: BTL, bilateral tubal ligation; COCs, combined oral contraceptives; D&C, dilation and curettage; EP, ectopic pregnancy; IUD, intrauterine device; OR, odds ratio (OR).

Note: * *p* < 0.05; Chi-squared test.

In consistency with the study design, no significant difference was observed between the two groups in terms of age, BMI, gestational age, EP size, and β-hCG values on admission ( *p* > 0.05). The mean amount of free peritoneal fluid before treatment in the ruptured group was of 58.44 ± 29.31 mm, and it was found to be significantly higher than the control group ( *p* = 0.000). The length of hospital stay was found to be significantly shorter in the ruptured group (9.44 ± 6.36 days versus 11.84 ± 7.14 days in the control group). There was no significant difference between the control and ruptured groups in terms of β-hCG values on admission (2,675.81 ± 2,288.22 versus 2,629.31 ± 2,047.12 mIU/mL respectively). The characteristics of the groups according to the obstetric and hematological parameters are presented in Table 2 . Upon assessing the CBC, there were no significant differences in Hb, PLT, PLR, MCV, RDW, and MPV values between the two groups ( *p* > 0.05); WBC was significantly higher in the control group (8,753 ± 2,435 × 1/µl versus 7,982 ± 2,271 × 1/µl in the ruptured group; *p* = 0.040), while the ruptured group presented significantly higher values for NLR (2.92 ± 0.86% versus 2.09 ± 0.6% in the control group; *p* = 0.000) and PDW (51 ± 9% versus 47 ± 13% among the controls; *p* = 0.019). There was a moderate positive correlation between NLR and the number of days until rupture in the case group (r = 0.585; *p* < 0.05), but no significant correlation was found with PDW (r = 0.381; *p* = 0.221).

**Table 2:** Obstetric and hematological features of the study groups

	Groups		Test statistic	*p*
	Control	Ruptured		
	Mean ± SD	Mean ± SD		
Age (years)	29.69 ± 5.55	29.71 ± 5.55	−0.021	0.983
Body mass index	25.06 ± 4.01	25.15 ± 4.65	−0.137	0.891
EP size (mm)	18.43 ± 7.64	19.34 ± 7.9	−0.736	0.463
Day of gestational age	42.33 ± 13.32	41.97 ± 13.4	0.157	0.875
Mean amount of free peritoneal fluid (mm)	27.1 ± 19.69	58.44 ± 29.31	−4.656	0.000*
Length of hospital stay (days)	11.84 ± 7.14	9.44 ± 6.36	2.225	0.027*
β-hCG on hospitalization (mlU/mL)	2675.81 ± 2288.23	2629.31 ± 2047.12	0.136	0.892
Hb (g/dL)	12.65 ± 1.14	12.79 ± 1.1	−0.788	0.432
WBCs (×1/µL)	8,753.49 ± 2,435.02	7,982.69 ± 2,271.04	2.074	0.040*
PLTs (×1/µL)	274,000 ± 56,013.5	257,756.41 ± 65,488.21	1.695	0.092
PLR (%)	132.56 ± 41.15	134.82 ± 40.18	−0.351	0.726
NLR (%)	2.09 ± 0.6	2.92 ± 0.86	−7.117	0.000*
MCV (fL)	85.87 ± 7.19	86.97 ± 5.56	−1.076	0.284
MPV (fL)	8.03 ± 0.86	8.04 ± 1.06	−0.105	0.917
PDW (%)	47.08 ± 13.17	51.28 ± 9.09	−2.366	0.019*
RDW (%)	14.32 ± 1.26	14.07 ± 1.25	1.277	0.203

Abbreviations: β-hCG, beta-human chorionic gonadotropin; EP, ectopic pregnancy; Hb, hemoglobin; MCV, mean corpuscular volume; MPV, mean platelet volume; NLR, neutrophil-to-lymphocyte ratio; PDW, platelet distribution width; PLR, platelet-to-lymphocyte ratio; PLTs, platelets; RDW, reticulocyte distribution width; SD, standard deviation; WBCs, white blood cells (leukocytes).

Note: * *p* < 0.05; *t* -test.

**Fig. 1:**
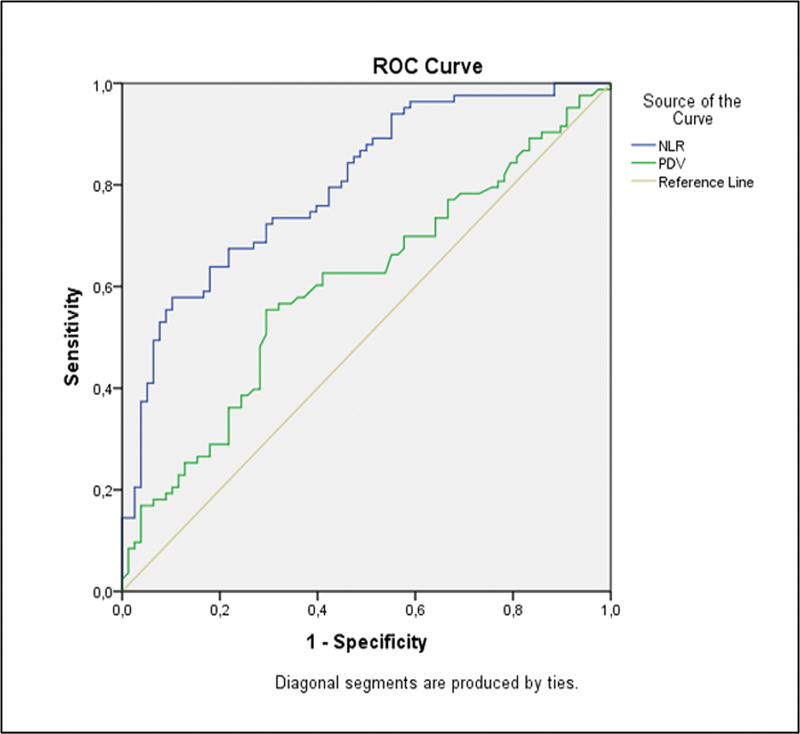
The receiver operating characteristic (ROC) curve for neutrophil-to-lymphocyte ratio (NLR) and platelet distribution width (PDW).

**Fig. 2:**
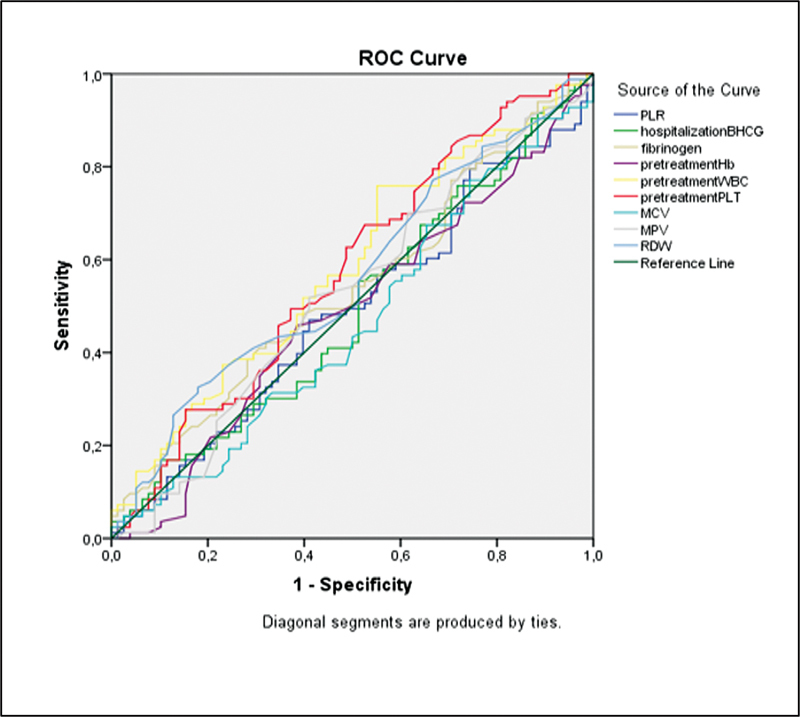
The ROC curve for other hematological markers.

The results of the ROC curve analyses of the hematological markers are shown in Table 3 . The AUC for NLR was of 0.800, as seen in Figure 1 , which is a significantly higher area ( *p* < 0.05), with 2.13% representing the optimal cut-off value. The AUC for PDW was of 0.601, as seen in Figure 1 , which is a significantly higher area ( *p* < 0.05), with 49.05% representing the optimal cut-off value. According to Table 3 and Figure 2 , no significant AUC was found regarding the values for β-hCG on hospitalization, WBC, Hb, PLR, RDW, MCV, and MPV.

**Table 3:** Results of the ROC curve analyses of hematological markers

	Area	Standard error	*p*	Asymptotic 95% confidence interval	
				Lower bound	Upper bound
NLR	0.800	0.034	0.000	0.733	0.867
PDW	0.601	0.045	0.026	0.514	0.689
β-HCG on admission	0.493	0.046	0.872	0.403	0.582
Hb	0.486	0.046	0.760	0.396	0.576
WBCs	0.584	0.045	0.064	0.496	0.672
PLTs	0.576	0.045	0.095	0.488	0.665
MCV	0.464	0.046	0.429	0.374	0.553
MPV	0.524	0.046	0.595	0.434	0.614
RDW	0.560	0.045	0.185	0.472	0.649
PLR	0.489	0.046	0.810	0.399	0.579

Abbreviations: β-hCG, beta-human chorionic gonadotropin; Hb, hemoglobin; MCV, mean corpuscular volume; MPV, mean platelet volume; NLR, neutrophil-to-lymphocyte ratio; PDW, platelet distribution width; PLR, platelet-to-lymphocyte ratio; PLTs, platelets; RDW, reticulocyte distribution width; ROC, receiver operating characteristic; WBCs, white blood cells (leukocytes).

Note: * *p <* 0.05; ROC curve.

## Discussion

The MTX therapy can be safely administered in the treatment of EP; unfortunately, it may fail, resulting in tubal rupture of the ampullary EP. However, many factors affect the failure of the MTX treatment, such as the general health of the ectopic pregnancy, 14 pretreatment levels of β-hCG, 15 EP size, 16 and gestational age, 17 but the values found or these variables were similar in the two groups in the present study. In addition, systemic inflammatory markers derived from the CBC can affect many parameters. Cho et al. 18 stated that hematological inflammatory markers such as NLR may be an indicator of the aging process, and obesity is associated with chronic inflammation. 19 However, in the present study, the ages and BMIs of both groups were similar, and patients with systemic comorbidities were also excluded. Although we ruled out all these variables that may affect inflammatory markers and rupture rate, we determined that one of the two groups with similar characteristics ruptured, while the other was cured. In the present study, we investigated whether CBC-derived inflammatory markers could be used to predict the failure of the MTX therapy and the resulting life-threatening EP rupture despite all of these similarities.

The history of PID was significantly higher in the control group, but the relationship between the history of PID and rupture is still inconsistent according to studies in the literature. 14 20 Similarly, the size of the pelvic effusion was not significant in terms of rupture in these studies, 14 20 and the size of the effusion was higher in the ruptured group in the present study. Similarly to the study by Cohen et al., 21 in the present study, the mean time between the beginning of the MTX and rupture was of 7 ± 3.19 days. In an attempt to predict rupture, studies have generally investigated the properties of EP. 14 21 22 However, in the present study, we aimed to investigate the relationship between CBC parameters routinely obtained from all hospitalized patients and the prediction of rupture of tubal EP undergoing the MTX treatment. There are several similar and recently-published; 23 24 25 26 however, the characteristics of the EP differ among the groups in these studies, such as baseline β-hCG levels. Nonetheless, parameters such as NLR, PLR, RDW, and MPV are as effective to the success of the MTX treatment as baseline β-hCG in these studies. 23 24 25 26

In the present study, the NLR and PDW were higher in the ruptured group compared to the control group, while the WBC was lower: 7982 ± 2271 × 1/µl versus 8753 ± 2435 × 1/µl respectively; *p* < 0.05. In the study by Kan et al. 23 with 142 patients, the ruptured group presented significantly higher values for NLR (4.62 ± 3.13 versus 2.67 ± 1.43; *p* < 0.05) and PLR (162.94 ± 63.61 versus 115.84 ± 41.15; *p* < 0.05). Moreover, Kanmaz et al., 24 who investigated the monitoring of hematological markers in 434 patients undergoing the MTX showed that, in addition to monitoring β-hCG levels in the group in which the treatment was successful, the NLR values, which were monitored on the 1st, 4th and 7th days after a single dose of MTX were lower ( *p* = 0.012; *p* = 0.035; and *p* = 0.001 respectively). However, in the aforementioned study, there was no significant difference between the groups in terms of PDW and PLT values on the 1st, 4th, and 7th days ( *p* > 0.05). 24 In the study by Cekmez et al. with 115 patients, the cut-off values for MPV and NLR were determined as 10.1 fL and 1.82% respectively, and they showed similar sensitivity and specificity in estimating the success of the MTX treatment. 25 In this study, in a homogeneous group, with a high AUC, the cut-off value for NLR was found to be of 2.3% and for PDW, of 49.05%. Additionally, Akkaya and Uysal 26 found that WBC, Hb, MCV, PLT, PLR, and PDW values were similar in the surgical and MTX groups, while RDW and MPV were significantly increased in the MTX group. In the present study, other hematological markers such as Hb, MCV, PLT, and PLR were found to be similar in both groups ( *p* > 0.05). Although a positive correlation between β-hCG values and the risk of rupture has been reported in many studies in the literature, a precise threshold for the risk of rupture cannot be determined; therefore, Galstyan and Kurzel., 27 in a study with 183 patients, did not report a reliable β-hCG value to predict the risk of rupture in EP. In the present study, there was a moderate positive correlation between NLR and the number of days since the beginning of the MTX treatment until rupture in the case group, but no significant correlation was found with PDW.

The decision for surgical treatment or medical management of an EP should be guided by a patient choice based on initial clinical, laboratory, and radiological data, as well as a discussion about the benefits and risks of each treatment. A clinical decision is made when an EP is ruptured, and its clinical features include: hemodynamic instability, pelvic pain, tenderness in the abdominal examination, signs of intraperitoneal bleeding, decrease in Hb values or the need for blood transfusion, and detection of intraabdominal fluid with coagulum by radiological imaging methods. According to the ACOG guidelines, the presence of intraabdominal fluid alone is not an absolute contraindication for MTX and is not sufficient for surgery. 3 In a study 28 on EP risk in patients with isolated free peritoneal fluid on transvaginal ultrasound, 42% of patients with isolated free peritoneal fluid were diagnosed with EP. Accordingly, if the fluid is echogenic or the volume is large, the risk of EP diagnosis increases; EP was detected in 22% of the patients with moderate anechoic fluid, and in 73% of the patients with large volumes of fluid or any echogenic fluid. 28 Patients with isolated free peritoneal fluid are at moderate risk of developing EP, but free fluid in the abdomen may represent the blood, serous fluid, or pus, and serous fluid is often found in asymptomatic healthy women. Moreover, in an ultrasound-based prediction model for the determination of the amount of hemoperitoneum in EP by Fauconnier et al., 29 tubal rupture was only detected in 40% of the cases. In this study, 29 peritoneal free fluid was detected at a rate of 21/78 (26.9%) in the MTX group, and of 34/83 (40.9%) in the ruptured group. However, in the present study, free peritoneal fluid, NLR, and PDW were found to be significantly higher in the ruptured group ( *p* < 0.05). The correlation of free peritoneal fluid with NLR and PDW is presented in Table 4 . A low positive correlation was only observed between free peritoneal fluid and NLR.

**Table 4:** Correlation of free intraabdominal fluid with NLR and PDW

		Free peritoneal fluid	Neutrophil-to-lymphocyte ratio (NLR)	Platelet distribution width (PDW)
Free peritoneal fluid (mm)	Pearson Correlation	1	0.373**	0.164
	Sig. (2-tailed)		0.005	0.233
	Sum of squares and cross-products	49,227.527	715.609	3,877.282
	Covariance	911.621	13.252	71.802
	N	55	55	55

Notes: *Correlation is significant at 0.05 (2-tailed); **correlation is significant at 0.01 (2-tailed).

In the present study, NLR and PDW were higher in the ruptured group, while WBC was higher in the control group. Extravasation of WBCs is an important feature of the inflammatory response, and Kan et al. 23 found that the WBC count for EP was significantly higher than for intrauterine pregnancies, suggesting that an increase in WBC count is associated with the development of EP. The higher increase in WBC in the control group in the present study suggests that it may have played an important role in suppressing the maternal immune response against a semiallogeneic fetus and limiting the invasiveness of the trophoblast. 30 However, the direction in which the increase is shifted (left or right) is more important in clinical use, rather than an increase in WBC count, because neutrophilia indicates systemic inflammatory events, whereas lymphopenia reflects an inadequate response in cellular immunity. 31 The reason why an increase in NLR indicates a poor prognosis is based on the mechanism by which neutrophils dominate and suppress cytotoxic T-cells through cytokines and chemokines in inflammatory processes. 31 In this case, the increase in the NLR is an indirect indicator of the immune response of he host, and it was found to be higher in the ruptured group in the present study. However, the underlying mechanisms for the relationship between inflammation and the outcome of the EP treatment need further study.

The strengths of the present study are that it was conducted with a homogeneous group (the groups were similar in terms of age, BMI, gestational age, baseline β-hCG, and EP size), with the exclusion of factors influencing systemic inflammation, at a tertiary center. The limitation of the study is its retrospective design. In conclusion, we can state that systemic inflammatory markers obtained from CBC are convenient and affordable tools to establish the difference in MTX response in patients with similar characteristics and in estimating the risk of tubal rupture in EPs.

## Conclusion

The present study shows that systemic inflammatory markers such as NLR and PDW may be suitable tools to detect the risk of tubal rupture during the MTX treatment for EP. These markers can help physicians estimate which cases will or will not benefit from the treatment right at the beginning. We think these systemic inflammatory markers, which are easily obtained from the CBC without additional cost, should be evaluated and considered in larger cohort studies.
